# Event and Comment

**Published:** 1916-07

**Authors:** 


### DENTAL REGISTER

Vol. LXX JULY, 1916 No. 7

### EVENT AND COMMENT

SOCIAL IDEALS. This is the season when a large number of newly graduated dentists are seeking suitable locations for practice. The important requirements in the average is that there is a field not over-crowded and a promise of a quick recognition by the community for the worth and skill of the new dentist. There are all grades of skill to be found in these men. One plans to serve merely for the monetary recompense that is customary; another locates in the community where he can render the better class of service to needy and appreciative sufferers. We often wonder if the young graduates think of their efforts in ministration in the light of a call to useful social service; and, for that matter, does the average practitioner know that he is filling a most important place in society, and that much of the community's comfort and prosperity is dependent on his faithfulness? There is so much being said and written in these days about "business building" and "selling services" that there is danger that the main issues may be overlooked in the scramble to keep up with the ever-increasing demands for a more extravagant living. It is a well-known fact that there is at the present time an insufficient force of practitioners to render all the service demanded by the people, and much less is there an opportunity to do any aggressive work for the careless and ignorant who do not appreciate the necessity of healthy teeth. In the light of modern research we are coming to realize the importance of good teeth on general physical health, to the extent that health officers and school boards are making examination and treatment of poor children's teeth an important part of their activities. All of this is leading directly to the next important step, which is a proper conception of the social bearing of good teeth on good health and the common welfare of people who are compelled to live in more or less proximate contact. Without discussing the matter at length, we feel justified in making the statement that there is no more universal method of spreading disease than is afforded by diseased conditions of the mouth and throat. Dentists have long recognized this fact, and the medical men are gradually coming to take some notice of oral affections, but the people who are most directly interested need to be made intelligent on a subject of such vital concern. Health boards are trying to control the more apparent dangers by stopping spitting in public places and abolishing the roller towel and the common drinking cup, but how shall they stop the contamina tion of the air in our homes and places of public assembly from coughing and sneezing, and even from breathing and talking through mouths that are very cesspools of filth? We are forced by our educational methods, our business relations and even our social relations to come into such close contact with each other as to make each of us his brother's keeper, or, as most generally happens, his menace. We do not exactly know how all this is to be remedied, as we are not prepared to suggest social segregation, but is it not possible for us to keep this thought always in mind in the work which we are called upon daily to perform, in order that we may get higher ideas of the importance and responsibility of our services to the entire public as well as to the individuals whom we directly serve? Let us do all we can to educate those with whom we come in contact to the dangers that threaten, and encourage every effort to advance public propaganda of every kind that makes for better oral health. We shall begin then to realize the necessity for our calling and command the respect which shall justify our claim for professional recognition; and, better yet, we shall have the conscious joy of spending our lives in helping to bring the reign of happiness.

---

MEMORIAL TO DR. H. A. SMITH. During the commencement exercises of the Ohio College of Dental Surgery a fine memorial tablet of Dr. H. A. Smith was placed in the college building. The tablet is of bronze and records the dates of the birth and death of Dr. Smith and his relation as Dean and Professor of Operative Dentistry. In the center is a bas-relief which is an excellent picture of Dr. Smith. This is an appropriate and excellent method of making durable records of the life and works of men who have given their best efforts for the advancement of worthy ideals. Its constant presence before the student body should inspire others to noble endeavors and unselfish living.

